# A validation study of the LABIRINTO scale for the evaluation of autism spectrum disorder in children aged 2 to 4 years

**DOI:** 10.47626/2237-6089-2020-0141

**Published:** 2021-12-10

**Authors:** Milena Pereira Pondé, Daniele de Brito Wanderley, Laise Dodô de Menezes, Fernanda Lima Gomes, Gustavo Marcelino Siquara

**Affiliations:** 1 Laboratório Interdisciplinar de Pesquisa em Autismo Escola Bahiana de Medicina e Saúde Pública Salvador BA Brazil Laboratório Interdisciplinar de Pesquisa em Autismo (LABIRINTO), Escola Bahiana de Medicina e Saúde Pública, Salvador, BA, Brazil.; 2 Universidade Federal da Bahia Salvador BA Brazil Universidade Federal da Bahia, Salvador, BA, Brazil.; 3 Escola Bahiana de Medicina e Saúde Pública Salvador BA Brazil Escola Bahiana de Medicina e Saúde Pública, Salvador, BA, Brazil.

**Keywords:** Autism, diagnostic evaluation, psychometrics, validation

## Abstract

**Objective:**

To find evidence of the content, construct, and criterion validity of the LABIRINTO scale for the diagnosis of autism spectrum disorder (ASD) in children aged 24-59 months.

**Methods:**

The scale was constructed in four stages: 1) items were defined based on an extensive literature review and discussions with autism and child development specialists; 2) child development specialists evaluated each item; 3) a preliminary version of the scale was applied to children diagnosed with ASD to enable any necessary adjustments; 4) the scale was then applied to 27 children with typical development and no neurodevelopmental disorder and 48 children with ASD. According to the 5th edition of the Diagnostic and Statistical Manual of Mental Disorders (DSM-5) and the Childhood Autism Rating Scale (CARS), clinical diagnosis constitutes the gold standard.

**Results:**

The scale’s psychometric indexes were appropriate for construct validity, with Kaiser-Meyer-Olkin = 0.94 and root mean square error of approximation = 0.000. Only one factor on the scale had a Cronbach alpha of 0.97. The receiver operating characteristic curve indicated a cutoff of 12, with a sensitivity of 100% and specificity of 100% for distinguishing children with ASD from those with typical development.

**Conclusion:**

This study confirmed the validity of the LABIRINTO scale.

## Introduction

Several instruments are used in clinical practice and in research to evaluate children, adolescents and adults with suspected autism spectrum disorder (ASD). The classifications of the American Psychiatric Association’s Diagnostic and Statistical Manual of Mental Disorders (DSM) and those of the World Health Organization (WHO)’s International Classification of Diseases (ICD) indicate diagnostic criteria for mental disorders that serve as a reference for clinical evaluation in psychiatry and are considered the gold standard for diagnosis.^1,2^ The fifth revision of the DSM (DSM-5) defines ASD based on two broad criteria: persistent difficulties with social communication and social interaction; and restricted and repetitive patterns of behaviors, activities, or interests.^3^ Also, the severity of the symptoms is classified as mild, moderate, and severe, according to the degree of impairment that the symptoms cause to the patient’s adaptive functioning and their need for support.^3^

An experienced clinician’s classic medical evaluation through anamnesis, physical examination, and psychiatric examination is considered the gold standard for any medical diagnosis.^4^ Diagnostic manuals, in conjunction with the examiner’s clinical skills, serve to determine the presence of all the symptoms required for an individual to be classified as having or not having any given disease. In practice, the symptoms of many mental disorders overlap, often making it difficult to perform a differential diagnosis. The most widely accepted instruments for this purpose worldwide are the Autism Diagnostic Observation Schedule (ADOS) and the Autism Diagnostic Interview-Revised (ADI-R). Portuguese versions of both of these instruments already exist but are not yet available for use.^5,6^

The ADI-R is a semi-structured interview to be applied to parents regarding the child’s development between the ages of 4 and 5 years. Because it is an interview and does not involve direct assessment of the child, it has widely recognized limitations.^7^ The main limitations refer to the nature of the instrument: the assessed life span refers to the age of 4 to 5 years, not applying to younger children; for children over 5 years of age, the assessment relies on the parents’ memory bias; finally, it is based on parents’ perception rather than on a direct evaluation of the child.

In the first version of ADOS, the complete instrument encompassed the three core domains of the disorder. However, the instrument’s algorithm (which allows patients to be classified) does not include symptoms associated with repetitive and ritualistic behavior.^8^ This criterion might lead to the inclusion of children currently classified by the DSM-5 as having a social communication disorder. Indeed, the DSM-5 diagnostic criteria require a more significant number of symptoms for a diagnosis of ASD to be established than the previous versions of the manual, e.g., DSM-IV-TR. Therefore, patients who met the criteria for pervasive developmental disorders in that classification may not be classified as ASD according to DSM-5 criteria.^9^

The second version of the instrument, ADOS-2, was made in accordance with the DSM-5 classification, which also requires the presence of symptoms of rigid and repetitive behavior for the diagnosis of ASD.^10^ To be classified as having autism or ASD using ADOS-2, the sum of the scores defined in the algorithms of the four modules must reach the minimum cutoff points in the domains of communication, reciprocal social interaction, and restricted and repetitive behavior.^10^ Sensitivity, specificity, and reliability of the ADOS-2 for a diagnosis of ASD have been demonstrated for both the original version in English and versions in other languages.^11,12^

Within the context of ADOS-2, there are some aspects that we consider essential to be advanced. First, most items in ADOS-2 have only three response options, the first one corresponding to the typical child’s response, plus only two variations of response to account for the broad spectrum of autism. Despite facilitating the instrument’s use, the reduced number of options does not allow a more nuanced assessment of the severity of each ASD symptom independently. Second, the application of ADOS-2 involves high costs with training and acquisition of the application material. Even if the instrument were available for use in Brazil, the high cost could limit its use in many clinical settings. Third, the modules are divided by level of access to verbal communication, making it challenging to apply the instrument to adults that are non-verbal or minimally verbal. This happens because the activities proposed for the pre-verbal/single words module are not adapted for non-verbal or minimally verbal adolescents and adults, but rather, specifically for younger children. Forth, the birthday party activity is not part of the cultural context of children from some non-Western cultures and children from some religious backgrounds, such as Jehovah’s Witness families. This aspect makes it difficult to carry out that activity in these groups, even though it is essential to assess symbolic play.^13^

The Childhood Autism Rating Scale (CARS) is a short, essentially observational scale applied to children aged 2 to 18 years.^14^ CARS has been translated and validated for use in Brazilian Portuguese.^15^ The original cutoff points of this version of the CARS exclude milder cases of autism, which is a disadvantage. Some studies show that the CARS cutoff excludes the mildest and even moderate cases of the autism spectrum and have suggested establishing lower cutoff points to include the diagnosis of milder cases of ASD, previously referred to as pervasive developmental disorder-not otherwise specified (PDD-NOS).^13-16^ Other drawbacks include the fact that evaluating the core symptoms of autism is superficial, and the instrument’s application has not been standardized.

The Interdisciplinary Research Laboratory for Autism (Laboratório Interdisciplinar de Pesquisa em Autismo [LABIRINTO]) developed a diagnostic instrument that considers different activities and questions according to different age groups. The LABIRINTO scale for the diagnosis of autism in children aged 2 to 4 years and 11 months is part of a project that includes the development of diagnostic instruments that enable the diagnostic evaluation of autism at different life stages in addition to detailing frequently associated symptoms. Each item on the LABIRINTO scale for the diagnosis of autism assesses one of the main symptoms for the diagnosis of ASD. Each item/question has five alternative responses (quoted from 1 to 5), which indicate different levels of impairment of the assessed symptom; the item quoted as 0 (zero) corresponds to typical development. The complete project of instruments includes the module presented in this article, which assesses core symptoms of autism in children aged 2 to 4 years and 11 months; plus a module that evaluates children from 5 to 7 years and 11 months; another module that evaluates verbal persons over 8 years; and a last module that evaluates non-verbal persons over 8 years old. For each of these modules, a session assesses associated symptoms often found in patients with ASD, such as opposing behavior, behavioral outbursts, obsessive-compulsive symptoms, aggressive behavior, hyper- and hypoactivity. The project also includes two self-response instruments to be completed by parents: one assessing symptoms of sensory hyper- and hyporesponsiveness; and another assessing eating behavior, which has already been validated and is available for use.^17^

The objective of the present study was to find evidence of the content, construct, and criterion validity of the LABIRINTO Autism Diagnostic Scale in the module designed to evaluate children aged 2 to 4 years and 11 months.

## Methods

### First stage: content validity

To create the scale, the constructs that are important for a diagnosis of ASD were identified based on its symptom triad and on relevant clinical symptoms commonly associated with autism. The scale was developed following a thorough review of the literature and debates with experts/specialists in autism and child development. The team working in this first stage consisted of a psychiatrist, a psychologist, a speech therapist, and a pediatric neurologist. All of them had over 10 years of clinical experience in evaluating and treating children, adolescents, and adults with ASD and other neurodevelopmental disorders.

The first version of the instrument was sent to 10 specialists with expertise in different areas of ASD and child development (psychiatry, neurology, psychology, speech therapy, pedagogy, and occupational therapy) to evaluate the construct and provide suggestions. A letter was sent along with that first version of the instrument asking the professionals to inform whether they considered that the instrument’s items were adequate for the constructs that they represented and to suggest modifications accordingly. Five specialists answered: a pediatric neurologist, a child psychiatrist, a speech therapist, an occupational therapist specialized in sensory integration, and a psychologist with extensive training in child development. There was an agreement regarding most of the items; however, changes were also suggested and were, in general, incorporated following debates with the specialists.

In 2017 and 2018, the instrument was applied to 56 patients aged 2 to 26 years who had previously been diagnosed with ASD. The objective was to test the instrument’s applicability and the adequacy of the proposed items in the practice of diagnostic evaluation. In that step, the authors observed that the possible responses for some items did not meet the characterization of some of the patients. Therefore, the response options for various items were changed at that moment to take the diversity of the patients evaluated into consideration.

The present paper describes the construct and criterion validity processes of the scale version used to evaluate children aged 24 to 59 months.

This study was approved by the internal review board of Escola Bahiana de Medicina e Saúde Pública (CAAE 00467217.2.0000.5544) and followed the regulations defined under Resolution 466/12 of the Brazilian National Health Council for research involving human beings. All participating parents/guardians gave their written consent.

### Second stage: construct and criterion validity

For construct validity, exploratory factor analysis was conducted to obtain psychometric indexes. Criterion validity was evaluated by analyzing the degree of accuracy of the scale in predicting a diagnosis of autism in the proposed age group. Therefore, diagnosis was the criterion to be evaluated in relation to the score obtained from applying the scale. This stage in the validation process considered the clinical diagnosis of autism as defined in the DSM-5 and used the CARS as a reference.^3-13^ In both cases, the diagnosis was made by the same team, consisting of a psychiatrist and a psychologist.

### Sample

Autistic children were referred by a clinic specialized in the diagnostic evaluation of autism. In contrast, children with typical development were selected among those attending a municipal nursery. The sample consisted of 75 children: 48 with a diagnosis of ASD and 27 with typical development. Table 1 shows the age distribution of the children in the two groups and the severity of symptoms in children with a diagnosis of ASD (Table 1).

**Table 1:** Age in children with ASD and typical development and severity of symptoms in children with ASD

	Age Mean (SD)	Severity of ASD symptoms n (%)	2 years n (%)	3 years n (%)	4 years n (%)
Typical development (n = 27)	3.43 (0.63)	-	2 (7.1)	12 (42.9)	13 (50.0)
ASD (n = 48)	2.89 (0.86)	-	20 (41.6)	12 (25.5)	15 (31.9)
Severity of ASD					
Mild		20 (41.7)			
Moderate		21 (43.8)			
Severe		7 (14.5)			

ASD = autism spectrum disorder; SD = standard deviation.

### Evaluation instruments

#### LABIRINTO scale

The LABIRINTO scale consists of a list of structured activities to be performed with the child. Each evaluation lasts on average 40-60 minutes. As described in the instrument, specific toys and games are used to evaluate the symptomatic domains covered by the scale. After the interaction, the items on the scale should be completed according to the child’s response. The scale consists of 15 items that evaluate the degree to which the behaviors associated with the symptoms of ASD are affected. The following domains are assessed: social interaction (3 items), verbal communication (3 items), non-verbal communication (5 items), and restricted and repetitive behaviors (4 items).

In the social interaction domain, the items evaluate the following aspects: child’s response to being approached and response to the games and play proposed by the evaluator; attempts to make eye contact with others, which evaluates the attempts made by the child during the entire evaluation, both with the examiner and with his/her parents (or caregivers present); and social smiling, which refers to smiling as a means of social interaction. In the verbal communication domain, the items evaluate: the quality of expressive verbal communication, or the quality of verbal expression, including the presence of abnormalities such as echolalia, changes in prosody and pronominal inversion; quality of linguistic repertoire, referring to verbal communication skills, including linguistic repertoire and the ability to communicate using this repertoire; and reciprocity in verbal communication, i.e., reciprocity established in dialogue. The items in the non-verbal communication domain assess the child’s response to being called by his/her name, or whether the child looks towards the person when called; visual contact, which evaluates the use of eye movement as a communication aid; intention of joint attention, i.e., whether the child’s gaze follows to indicate to the evaluator his/her interest in an object; response to joint attention, which evaluates whether the child’s gaze follows the evaluator’s eye movement or gestures to indicate an object out of reach of both; and gestures in communication, or the use of hand and facial gestures as communication aids. Finally, the items referring to restrictive and repetitive behaviors are: playing/symbolization, which evaluates the quality of the child’s play; difficulty with change/rigidity, or the child’s fixation on a specific activity; repetitive/stereotyped movements, which concerns the presence of these movements; and restricted interests, i.e., whether there is a particular insistence or idiosyncrasy in the use of objects. The items are presented on a scale with scores 0 to 5, where 0 corresponds to the behavior of a typical child and the other scores (1-5) refer to levels of severity concerning the abnormality presented by the child in the item evaluated.

#### Childhood Autism Rating Scale (CARS)

CARS is a 15-item scale that helps identify children with ASD, distinguishing them from children without autism but with developmental disabilities, and differentiating between mild-to-moderate autism and the severe form of the disorder.^13-18^ It is a blunt instrument that can be used with any child over 2 years. A trained clinician observes the child’s behavior over 15 domains that are often affected in autism: relationship to people, imitation, emotional response, body use, object use, adaptation to change, visual response, listening response, taste-smell-touch response, fear or nervousness, verbal communication, non-verbal communication, activity level, level and consistency of intellectual response, and general impressions. The examiner assigns scores ranging from 1 to 4 for each item, where 1 indicates appropriate behavior for age and 4 indicates severely abnormal behavior. The total number of points in the CARS ranges from 15 to 60, with scores below 30 indicating that the individual is not autistic. In contrast, a score of 30 to 36.5 suggests mild to moderate autism, and a score of 37 to 60 indicates severe autism.^13^ Some studies suggest establishing lower cutoff points to include the diagnosis of milder autism spectrum disorders (previously referred to as PDD-NOS). The Japanese version of the CARS (CARS-TV) suggests cutoff points of 25.5/26 to differentiate individuals with PDD-NOS from those with mental retardation.^14^ A study conducted with Caucasian children suggested a cutoff point of 25.5 to differentiate 4-year old children with PDD from those without PDD.^19^ A cutoff point of 20/21 was proposed to identify milder cases of ASD in a population of immigrants.^15^ The CARS has been translated and validated for use in Brazilian Portuguese.^16^

## Anamnesis

A detailed anamnesis was conducted with parents to investigate aspects of their children’s development, with the parents providing information on the principal issues that they had noticed and answering specific questions on the child’s development of verbal and non-verbal communication, social interaction with adults and with peers, restrictive and repetitive patterns of behavior, interests and activities, behavior at school, and the presence of sensory symptoms.

## Diagnosis according to the DSM-5

The diagnosis of ASD is fundamentally clinical and is based on child observations, interviews with the parents, and application of specific instruments.^20^ Based on the child’s evaluation and the anamnesis performed with the parents, the child’s diagnosis is made following DSM-5 criteria.^3^ The same psychiatrist performed all the diagnoses, in most cases working together with a psychologist and a team consisting of resident doctors and psychology students.

## Procedure

Children with a suspected diagnosis of autism were referred by the Bahia Association of the Friends of Autistic Individuals (Associação de Amigos do Autista da Bahia) for diagnostic evaluation. The evaluations were made at the psychology clinic of Escola Bahiana de Medicina e Saúde Pública, and the parents signed an informed consent form allowing the evaluation data to be used anonymously for the specific purposes of this study. The child was evaluated using the activities described in the evaluation manual of the LABIRINTO scale. Previously instructed trainee psychologists and medical residents in psychiatry performed the evaluation, supervised by a psychiatrist and a psychologist who monitored the process. The same team performed anamnesis. The evaluation lasted, on average, 90-120 minutes. After this evaluation, a team consisting of the supervising psychiatrist and psychologist and the psychology trainees and resident doctors met to discuss the evaluation, complete the instruments, and define the diagnosis per the DSM-5 criteria.

Children unsuspected of having developmental disorders were referred by their teachers and evaluated by previously trained psychology or medical students in a private room at the school. The evaluation was performed using the activities described in the manual of the LABIRINTO scale. The parents signed an informed consent form allowing the evaluation data to be used anonymously for this research study. Once the evaluation was through, the trainees completed the CARS and the LABIRINTO scale. If any sign of a developmental disorder was detected, the child was excluded from the sample and referred to the evaluation team.

## Data analysis

To evaluate the evidence of validity based on the instrument’s internal structure, exploratory factor analysis was performed using the FACTOR software program version 9.23.^21^ The analysis was based on a polychoric correlation matrix using the robust diagonally weighted least squares (RDWLS) as the extraction method.^22^ The decision regarding the number of factors to be retained was made using the parallel analysis technique with a random permutation of the data found.^23^ This technique was chosen because of the ordinal response measurement level that is characteristic of the instrument’s responses, the violation of the hypothesis, and the data’s multivariate normality.^24^ Therefore, the robust unweighted least squares (RULS) extraction method was used, which is considered appropriate for data with a normal or non-normal distribution and has been found to perform well in databases with many items.^25^ For factor retention, the parallel analyses method (optimal implementation of parallel analysis) was used.^26^

The goodness-of-fit of the model was evaluated using the root mean square error of approximation (RMSEA), the comparative fit index (CFI) and the Tucker-Lewis index (TLI). RMSEA values should be below 0.08, and CFI and TLI values should be over 0.90, or preferably, over 0.95.^27^

The JASP software and the Statistical Package for the Social Sciences (SPSS) were used to perform descriptive analyses. Pearson’s correlation and the Mann-Whitney test were used in the statistical analysis. The method established by Delong et al.^28^ was used to analyze the receiver operating characteristic (ROC) curve to obtain the instruments’ specificity and sensitivity. A ROC curve represents the proper positive fraction as a function of the false positive fraction at different cutoff points. To perform this calculation, the MedCalc statistical software program was used.

## Results

The process performed to validate the LABIRINTO scale is presented in two stages: construct validity, which describes the exploratory factor analysis performed; and criterion validity, which establishes scores of sensitivity and specificity to differentiate cases of autism from non-cases, i.e. children with typical development.

Based on the exploratory factor analysis, the optimal implementation of the parallel analysis method^23^ suggests one factor as being the most representative solution of the data: Kaiser-Meyer-Olkin (KMO) = 0.94247; Bartlett’s test of sphericity = 796.3; df = 105; p < 0.001; RMSEA = 0.000; non-normed fit index (NNFI) = 1.259; CFI: 1.126. Some indexes suggest that the scale should be used as a unidimensional factor: the evaluation index of the calculation of unidimensional congruence (UniCo) was 0.98 and the explained common variable (ECV) was 0.93. According to Ferrando & Lorenzo-Seva,^21^ when the UniCo is above 0.95 and the ECV is above 0.85, the data are considered unidimensional. Both scores, therefore, indicate that the scale is unidimensional. The robust goodness-of-fit indexes had an RMSEA = 0.000. A Cronbach’s alpha of 0.97 was found, suggesting excellent reliability of the LABIRINTO scale items.

The parallel analysis suggested one factor as being more representative of the data. The factor indexes are shown in Table 2. Only one of the factors had to be retained, since this one factor from the true data accounted for a percentage of explained variance greater than the random data. The items’ factor loadings are listed in Table 3, showing that the majority of the items had a factor loading above 0.800. The factor loadings represent the correlation between the indicator and the factor extracted; hence, factor loadings above 0.70 indicate a well-defined structure, with this being the goal of any factor analysis.^29,30^

**Table 2:** Results of the number of factors based on parallel analysis

Factors	Percentage of variance explained by the true data	Percentage of variance explained by random data (95%CI)
1	79.1621	16.8748
2	6.2149	14.4175
3	3.6185	12.7178
4	2.7958	11.2008
5	1.6663	10.0753
6	1.3957	8.9064
7	1.2586	7.9614
8	1.1910	6.9977
9	0.8317	6.1497
10	0.7206	5.2095
11	0.5356	4.4931
12	0.3169	3.5610
13	0.2458	2.7429
14	0.0465	1.8565

95%CI = 95% confidence interval.

**Table 3:** Factor analysis of the LABIRINTO scale

Item	Factor loading
1	0.914
2	0.930
3	0.868
4	0.900
5	0.858
6	0.929
7	0.933
8	0.908
9	0.922
10	0.940
11	0.939
12	0.891
13	0.695
14	0.823
15	0.690

The bivariate Pearson correlation found between the LABIRINTO scale and the CARS was r = 0.95 (p < 0.001), which is considered a strong correlation; hence, the total score in the two instruments tends to vary similarly, indicating criterion validity. There was a statistically significant difference in the mean scores obtained in the group of children with ASD compared to the group of children with typical development, both in the CARS (p < 0.001) and in the LABIRINTO scale (p < 0.001) (Table 4). This finding indicates that both scales were able to differentiate between these two groups of children. In the LABIRINTO scale, statistically significant differences were found in the mean scores obtained between the two groups of children for the subscales social interaction, verbal communication, non-verbal communication, and restricted and repetitive behaviors (Table 4), showing that the four subscales were able to differentiate between the two groups of children in a statistically significant manner.

**Table 4:** Comparison of scores between the LABIRINTO scale and the CARS scale

Instrument/group	N	Mean	SD	Mann-Whitney test
Total CARS score				
TD	27	16.48	2.88	< 0.001
ASD	48	50.67	12.30	
Total LABIRINTO score				
TD	27	2.85	3.67	< 0.001
ASD	48	37.1	12.92	
LABIRINTO subscales				
Social interaction				
TD	27	0.37	0.74	< 0.001
ASD	48	6.95	2.98	
Verbal communication				
TD	27	1.22	1.94	< 0.001
ASD	48	9.58	3.24	
Non-verbal communication				
TD	27	0.48	1.74	< 0.001
ASD	48	13.43	5.72	
Restrictive and repetitive behaviors				
TD	27	0.77	1.12	< 0.001
ASD	48	6.52	2.66	

ASD = autism spectrum disorder; SD = standard deviation; TD = typical development.

Considering the clinical diagnosis reached in accordance with DSM-5 criteria and the scores obtained in the LABIRINTO scale, an ROC curve was calculated for the group with ASD and the group with typical development in order to select the most appropriate cutoff point. This curve indicates the different cutoff points of the test or score according to their levels of sensitivity (Y axis) and specificity (X axis).^31^

According to the criteria established by Sweet & Picket (1982), cited in Muratori et al.,^32^ the area under the curve (AUC) is interpreted as follows: AUC < 0.7 suggests that accuracy is low, while an AUC of 0.7 to 0.9 indicates moderate accuracy and an AUC ≥ 0.9 indicates excellent accuracy. The area under the curve for the LABIRINTO scale was 0.99, with a significance level of p < 0.001. As shown in Table 5, when using a cutoff point of 12 for the presence of ASD according to the LABIRINTO scale, sensitivity resulted 100% and specificity 100% in relation to the gold standard, which is the clinical diagnosis in accordance with DSM-5 criteria.

**Table 5:** Cutoff points on the LABIRINTO scale, taking clinical diagnosis reached according to DSM-5 criteria as reference to differentiate cases from non-cases

Cutoff point	Sensitivity	Specificity
5	100	74
6	100	74
7	100	81
8	100	85
9	100	89
10	100	89
11	100	92
**12**	**100**	**100**
13	100	100
14	95.8	100
15	93.7	100
16	93.7	100
17	91.7	100

DSM-5 = 5th edition of the Diagnostic and Statistical Manual of Mental Disorders.

ROC curves were calculated based on the LABIRINTO subscales. This criterion is important in that it enables the minimum score for each one of the subscales to be presented in order to calculate the algorithm for diagnosis. The cutoff points calculated were as follows: for social interaction, over 2 points; for verbal communication, over 3 points; for non-verbal communication, over 1 point; and for restricted and repetitive behaviors, over 3 points (Table 6).

**Table 6:** Area under the receiver operating characteristic curve, sensitivity, specificity and cutoff point for each LABIRINTO subscale

LABIRINTO scale	AUC	Sensitivity (%)	Specificity (%)	Cutoff point
Social interaction	0.98	95.8	100	> 2 points
Verbal communication	0.98	95.8	88.9	> 3 points
Non-verbal communication	0.99	100	96.3	> 1 points
Restrictive and repetitive behaviors	0.98	100	89.6	> 3 points

AUC = area under the curve.

## Discussion

The present study aimed to find evidence of content, construct and criterion validity of the LABIRINTO scale for the diagnosis of autism. At the current stage of knowledge, diagnostic assessment tools for ASD must indicate, in addition to the central core symptoms of autism, the severity of these symptoms, and how that severity varies across the broad spectrum of autism. The LABIRINTO scale seeks to present five variations of severity for each symptom assessed, allowing to differentiate between more refined levels of severity for each symptom. This aspect is essential to identify, in addition to the diagnosis, the symptoms and symptomatic dimensions (verbal and non-verbal communication, interaction, and repetitive and restricted behavior) that need priority intervention, and to indicate the distribution of symptom severity in the broad spectrum of autism. Further refinement concerning each symptom’s severity spectrum will also help in the search for more specific biological indicators associated with each symptom or group of symptoms.

The validation procedures indicated that adequate factor indexes were found, with a correlation between the items and the unidimensionality of the LABIRINTO scale.^25,33,34^ Data from the exploratory factor analysis suggested evidence of validity between the items of the instrument. The reliability index was adequate, with a very satisfactory Cronbach’s alpha. Evidence of convergent validity was also found, with a high correlation between the LABIRINTO scale and the gold standard instruments for evaluating autism (CARS and DSM-5 criteria). Finally, the ROC curve calculated to evaluate criterion validity revealed the instrument’s excellent capacity to distinguish between the group of children with ASD and the group of children with typical development. The cutoff of 12 differentiates children with ASD from those with typical development, with sensitivity, specificity, positive predictive value, and negative predictive values of 100%.

In order for a diagnosis of ASD to be established according to DSM-5 criteria, abnormality has to be observed in non-verbal communication and social interaction, as well as restricted and/or repetitive behavior. Consequently, in addition to reaching the overall cutoff of 12 points in the LABIRINTO scale, the social interaction subscale score must be > 2, the score for the non-verbal communication subscale must be > 1 and the score for restricted and repetitive behaviors must be > 3. Therefore, the module for evaluating children aged 2 to 4 years and 11 months with suspected autism in the LABIRINTO scale is available for use in the Brazilian population: researchers and clinicians may apply it. However, a prerequisite for its use is certification in the use of the scale by the evaluator, i.e., completion of training given by the authors to ensure that the instrument is correctly applied and the items correctly completed.

## Trends in Psychiatry and Psychotherapy - Online-Only Supplementary Material


