# Supplementary material for: A validation study of the LABIRINTO scale for the evaluation of autism spectrum disorder in children aged 2 to 4 years
Source: Trends Psychiatry Psychother. 2021 Dec 10;43(4):320–8. doi: 10.47626/2237-6089-2020-0141 (PMC8835385; doi:10.47626/2237-6089-2020-0141)
Supplement: Supplementary file 1 [file 2238-0019-trends-43-04-0320-suppl01.pdf]

http://dx.doi.org/10.47626/2237-6089-2020-0141

LABIRINTO
Laboratório Interdisciplinar de Pesquisa em Autismo

Escala LABIRINTO para diagnóstico e caracterização clínica do TEA

Módulo – de 2 anos a 4 anos e 11 meses

Para responder adequadamente às questões da Escala LABIRINTO para diagnóstico e caracterização clínica do TEA, é necessário estar atento ao desenvolvimento típico da criança, bem como a avaliação por meio das tarefas sugeridas, o que vai proporcionar o preenchimento mais confiável do instrumento, esquivando-se de avaliações subjetivas e que levem em conta apenas a experiência prévia do avaliador. Após a avaliação da criança, reveja as informações sobre os marcos do desenvolvimento para responder com mais precisão à escala.

Sintomas Centrais

1. Interação social

1.1. Resposta da criança às aproximações feitas pelo avaliador

Esse item é avaliado com base nas observações feitas durante todo atendimento. O foco desse item são as respostas da criança às tentativas de aproximações feitas pelo avaliador, reveladas pelo engajamento (participar colaborando) da criança nos jogos e brincadeiras propostas pelo avaliador. A criança com autismo pode querer fazer a atividade somente do seu jeito, não colaborando com a proposta do avaliador, ou mesmo se opondo. Pode também fazer uma oposição passiva ao avaliador, que consiste em um comportamento de esquiva, que se revela com o não engajamento ou indiferença: por exemplo a criança prefere brincar sozinha longe do avaliador, fica de costas ou se afasta quando o avaliador se aproxima.

A criança fica indiferente a qualquer tipo de aproximação do avaliador.	5	
MUITO esforço é necessário para a criança aceitar a aproximação. A criança se engaja em UMA atividade proposta, que, em geral, representa o seu foco de interesse; E/OU se engaja apenas em brincadeiras de natureza física ou agitada.	4	
MUITO esforço é necessário para a criança aceitar a aproximação. A criança se engaja em POUCAS (duas ou três) atividades propostas. Inclui aqui também crianças que fazem a atividade apenas do seu jeito, ou faz o oposto do que o avaliador propõe.	3	
MODERADO esforço é necessário para a criança aceitar a aproximação. A criança se engaja em ALGUMAS (mais de quatro) atividades propostas.	2	
LEVE esforço é necessário para a criança aceitar a aproximação. A criança parece mais tímida no início, mas, após um leve esforço, aceita a aproximação. A criança se engaja na maioria das atividades propostas.	1	
A criança aceita bem a aproximação; se engaja rapidamente em todas ou na maioria das atividades propostas.	0	

1.2. Iniciativas feitas pela criança de buscar o outro

Esse item é avaliado com base nas observações sobre a busca de contato que a criança faz durante toda a avaliação, tanto a busca para com o avaliador como dos pais (ou cuidadores presentes). Considera-se que a criança busca o outro quando ela se dirige ativamente ao adulto para convidá-lo para participar de alguma brincadeira (oferece a bola para jogar o boliche, chama para fazer parte de um jogo simbólico), OU quando ela mostra ao adulto algum brinquedo ou algo que ela fez (um desenho, um encaixe, mostra que consegue abri a casa de chaves). A criança pode também buscar o adulto quando precisa de algo, como abrir o saco das peças de encaixe, pedir lanche, pedir para ir embora.

A criança EM NENHUM MOMENTO busca os pais/cuidadores e nem o avaliador.	5	
A criança busca o avaliador e/ou os pais/cuidadores APENAS para solicitar ajuda ou para fazer algum tipo de solicitação em seu próprio benefício.	4	
A criança busca o avaliador e/ou os pais/cuidadores APENAS UMA VEZ para participar de alguma brincadeira OU para mostrar algum brinquedo; OU crianças que buscam o adulto APENAS para provocar.	3	
A criança busca o avaliador e/ou os pais/cuidadores POUCAS VEZES (duas ou três) para participar de alguma brincadeira OU para mostrar algum brinquedo.	2	
A criança busca o avaliador e/ou os pais/cuidadores ALGUMAS VEZES (quatro ou cinco) para participar de alguma brincadeira E/OU para mostrar algum brinquedo.	1	
A criança busca o avaliador e/ou os pais/cuidadores para participarem de VÁRIAS atividades/brincadeiras. Mostra brinquedos aos pais e/ou avaliadores em VÁRIAS situações, indicando compartilhamento de algo prazeroso para si.	0	

1.3. Sorriso social

O sorriso social ocorre em resposta ao estímulo realizada por outra pessoa. A criança pode usar o sorriso como uma forma de aproximação social, nesse caso o sorriso não é apenas uma resposta, mas revela uma intenção comunicativa e de aproximação. Considerar que a criança pode sorrir sem relação com o contexto, nessas situações o sorriso não é social.

A criança NUNCA sorri; ou sorri APENAS sem relação com o contexto, como se sorrisse para si mesma.	5	
A criança sorri em resposta ao avaliador APENAS com brincadeiras muito agitadas ou de natureza física, como as de levantamento, giro ou cócegas; OU sorri APENAS quando os pais (cuidadores) fazem brincadeiras com as quais ela está habituada. Pode sorrir com mais frequência sem relação com o contexto, como se sorrisse para si mesma.	4	
A criança responde com sorriso às brincadeiras e iniciativas propostas pelo avaliador e NUNCA sorri como forma de buscar aproximação social.	3	
A criança responde com sorriso às brincadeiras e iniciativas propostas pelo avaliador. RARAMENTE (uma ou duas vezes) sorri como forma de buscar aproximação social.	2	
A princípio, a criança pode sorrir pouco, ou não sorrir. Após um tempo de aproximação do avaliador, a criança se mostra mais sorridente, de modo que ela passa a sorrir como forma de buscar uma aproximação social, assim como em resposta à situação ou ao sorriso do adulto.	1	

LABIRINTO
Laboratório Interdisciplinar de Pesquisa em Autismo

A criança responde ao sorriso social do avaliador, quanto sorri como forma de buscar uma aproximação social, não apenas em resposta à situação ou ao sorriso do adulto.	0	

2. Comunicação verbal

A resposta a esse item se baseia nos diálogos iniciados pelo avaliador e/ou pela criança.

2.1. Qualidade da linguagem verbal expressiva

Nesse item pesquisamos a presença de alterações na expressão verbal, que são características do autismo, sendo que algumas delas podem estar presentes em crianças menores, na fase de aquisição da linguagem. A inversão pronominal: a criança se refere a si mesma na terceira pessoa, podendo ocorrer mais comumente de 1,5 a 2,5 anos e eventualmente entre 2 anos e 6 meses e 3anos em crianças em desenvolvimento típico. Alteração discreta na prosódia consiste em acentuação aguda de algumas sílabas. Alteração marcada da prosódia: a fala da criança é claramente mecânica, com pouca variação entonacional. Não há variação entre padrões de fala afirmativo, interrogativo ou exclamativo. Não consegue ajustar a fala para diferentes interlocutores, ou seja, fala no mesmo padrão com bebês e adultos. Ecolalia tardia: a criança repete frases de filmes, de propagadas, caracterizando claramente que é uma frase copiada. A ecolalia tardia pode ser usada em contexto apropriado ou sem conexão com o contexto. Ecolalia imediata: a criança repete o que o adulto fala (ou faz uma repetição aproximada quando a linguagem verbal não é clara). Pode ocorrer como forma de processar a fala do interlocutor e tentar compreender, nesses casos, é mais comum em perguntas que utilizam 'quem', 'quando', 'onde' e 'como' ou perguntas abrangentes como 'o que você quer fazer?' Para melhor avaliação, prossegue a conversa com uma pergunta mais simples, se houver resposta, a repetição da criança não representa ecolalia. Exemplo: ["o que é isso?" complemente com: "É um carro, ou isso é uma bola?"], ["Quando você pode brincar?" complemente com: "Você pode brincar depois da escola?"]. Perguntas perseverantes ocorrem quando a criança pergunta repetidamente a mesma coisa, que em geral não tem relação com o contexto.

A verbalização da criança é exclusivamente ecolálica; OU perguntas perseverantes.	5	
Apresenta ecolalia imediata frequente; OU alteração marcada da prosódia; OU repete a mesma pergunta muitas vezes.	4	
Frequente ecolalia tardia com contexto adequado; OU ecolalia imediata eventual; OU alteração discreta de prosódia; OU repete a mesma pergunta eventualmente.	3	
De 2 a 3 anos: eventual ecolalia tardia com contexto adequado, a prosódia é próxima à esperada ou normal. De 3 a 5 anos: eventual ecolalia tardia com contexto adequado, a prosódia é próxima à esperada ou normal; OU apresenta inversão pronominal com frequência.	2	
De 2 a 3 anos: a criança fala com tom de voz muito baixo, com prosódia próxima à esperada ou normal. De 3 a 5 anos: a criança fala com tom de voz muito baixo, com prosódia próxima à esperada ou normal; OU apresenta inversão pronominal eventualmente.	1	
A criança não apresenta inversão pronominal, ecolalia, alteração na prosódia e nem perguntas perseverantes.	0	

LABIRINTO
Laboratório Interdisciplinar de Pesquisa em Autismo

2.2. Qualidade do repertório linguístico<br>*Esse item se refere ao domínio gramatical da língua, ou seja, o quanto a criança domina palavras e suas combinações em níveis linguísticos mais complexos.*		
<u>De 2 a 5 anos:</u> Não fala e/ou não emite nenhum som comunicativo, ou apenas grita ou emite grunhidos incompreensíveis; OU <u>De 4 a 5 anos:</u> APENAS articula sons de forma encadeada (silábicos), porém o conteúdo não é compreensível (como se falasse uma língua incompreensível – jargão).	5	
<u>De 2 a 3 anos</u>: APENAS articula sons de forma encadeada (silábicos), porém o conteúdo não é compreensível (como se falasse uma língua incompreensível – jargão). <u>De 3 a 4 anos:</u> fala palavras soltas, que NÃO comunicam; ou APENAS articula sons de forma encadeada, porém o conteúdo não é compreensível (como se falasse uma língua incompreensível – jargão). <u>De 4 a 5 anos:</u> fala POUCAS palavras soltas, que podem ou não comunicar.	4	
<u>De 2 a 3 anos</u>: fala palavras soltas, que NÃO comunicam. <u>De 3 a 4 anos:</u> fala POUCAS palavras soltas, que comunicam. <u>De 4 a 5 anos:</u> fala palavras soltas que comunicam e justapõe palavras, sem organizar frases gramaticais.	3	
<u>De 2 a 3 anos</u>: fala POUCAS palavras soltas, que comunicam. <u>De 3 a 4 anos</u>: fala palavras soltas que comunicam e justapõe palavras, sem organizar frases gramaticais. <u>De 4 a 5 anos</u>: fala frases com até 3 palavras. Começa a usar o pronome possessivo. Faz pequenos relatos de sua rotina.	2	
<u>De 2 a 3 anos</u>: fala palavras soltas que comunicam e justapõe palavras, sem organizar frases gramaticais. <u>De 3 a 4 anos</u>: fala frases com até 3 palavras. Começa a usar o pronome possessivo. Faz pequenos relatos de sua rotina. <u>De 4 a 5 anos</u>: fala frases com até quatro palavras, lembra e conta pequenas histórias.	1	
<u>De 2 a 3 ano</u>s: fala frases com até 3 palavras, começa a usar pronomes possessivos e usa muito o "Por quê?" Faz pequenos relatos de sua rotina. <u>De 3 a 4 anos</u>: fala frases com até quatro palavras, lembra e conta pequenas histórias. <u>De 4 a 5 anos</u>: tem um vocabulário bastante amplo. Em geral, fala muito. Expressa ideias e comenta sobre o seu cotidiano. Pode também fazer muitas perguntas.	0	

2.3. Reciprocidade na comunicação verbal<br>*A resposta a esse item se baseia nos diálogos iniciados pelo avaliador ou pela criança. Refere-se ao uso da fala com a intenção de se comunicar com as pessoas e de responder ao interlocutor. Um diálogo recíproco implica que ocorra alternância de fala entre os interlocutores sobre um determinado assunto: a criança inicia uma conversa, o avaliador responde e a criança comenta de volta; ou o avaliador inicia a conversa, a criança responde ou comenta, o avaliador comenta de volta e a criança responde ou comenta novamente.*		
Não usa a fala com intenção comunicativa e não atende a comandos verbais.	5	
Não usa a fala com intenção comunicativa, mas atende a alguns comandos verbais.	4	
É necessário muito esforço para que a criança responda ao que lhe é perguntado, as respostas são sempre muito curtas. Raramente se dirige verbalmente às pessoas de modo espontâneo, quando o faz, em geral, é para fazer alguma solicitação, ou pergunta sobre tema do seu foco de interesse.	3	

O diálogo é na base de perguntas e respostas: a criança responde às questões que o avaliador faz e faz perguntas ao avaliador, mas não sustenta um diálogo recíproco. OU fala sobre temas do seu interesse, mas quando o entrevistador tenta trazer outro assunto a criança retorna ao assunto do seu interesse.	2	
O diálogo recíproco ocorre em alguns momentos, sobretudo quando o tema interessa à criança, mas não é fluido como descrito no item “0”.	1	
O diálogo estabelecido com a criança é recíproco, a conversa flui de forma mútua. A criança faz perguntas e comentários, dando prosseguimento a uma conversa com turnos com o avaliador.	0	

3. Comunicação não verbal

3.1. Resposta ao chamado do nome.

A resposta a esse item se baseia nas respostas da criança às tentativas de chamada pelo nome que são feitas. Primeiro: o avaliador chama a criança pelo nome de forma clara por até quatro vezes. Segundo: caso ela não responda nenhuma das vezes, o avaliador pede aos pais/cuidador que chamem a criança pelo nome sem oferecer nenhum estímulo até três vezes. Terceiro: caso ela não responda, pedir que eles chamem pelo nome e apresentem algum estímulo (um brinquedo, ou algo que os pais saibam que atrai a criança), ou o próprio avaliador chama a criança mais uma vez pelo nome, mostrando algo que pode ser do interesse dela. Exemplo: “Fulano, olha o carrinho” (ou qualquer objeto que a criança mostrou predileção).

Não responde ao chamado do nome, nem mesmo quando um estímulo lhe é apresentado.	5	
Responde APENAS quando os pais/cuidadores OU o avaliador a chamam APRESENTANDO UM ESTÍMULO.	4	
Não responde ao chamado do avaliador, mesmo após quatro tentativas; responde ao chamado dos pais/cuidadores, SEM que apresentem um estímulo.	3	
Responde após três ou quatro tentativas ao chamado do avaliador, podendo ou não fazer contato visual direto com o avaliador.	2	
Responde, após duas tentativas, olhando diretamente para o avaliador ou responde imediatamente, porém sem fazer contato visual direto com o avaliador.	1	
Responde com contato visual direto quando o examinador a chama pelo nome na primeira tentativa.	0	

3.2. Contato visual com o avaliador.

Considera-se que quando duas pessoas interagem é muito frequente que se olhem enquanto conversam, de modo que o olhar auxilia a comunicação. Crianças com autismo comumente evitam o olhar, sendo esse um dos sinais precoces dessa condição. Esse item pontua o contato visual que a criança faz com o avaliador durante toda a avaliação.

A criança não faz contato visual com o examinador em nenhum momento.	5	
A criança olha para o avaliador por poucos segundos, em RARAS (uma ou duas) ocasiões.	4	

LABIRINTO
Laboratório Interdisciplinar de Pesquisa em Autismo

A criança olha para o avaliador por poucos segundos, em POUCAS (três ou quatro) ocasiões.	3	
A criança faz contato visual, em VÁRIAS (mais de cinco) ocasiões, por poucos segundos, sobretudo quando o avaliador a convoca para algo que a interesse, OU busca o olhar do avaliador APENAS quando quer chamar a atenção dele para algo que a interesse.	2	
A criança olha para o avaliador mesmo sem ser convocada, mas não por muito tempo e é menos frequente do que seria esperado.	1	
A criança mantém o olhar no examinador a maior parte do tempo em que estão interagindo, buscando o seu olhar em diversos momentos.	0	

3.3. Intenção de atenção compartilhada realizada pela criança

Esse item se refere às iniciativas que a criança tem de indicar ao avaliador (ou aos pais/cuidadores) o interesse dela em algum objeto, compartilhando com eles esse interesse. A atenção compartilhada, no desenvolvimento típico, consiste na criança olhar para o avaliador, olhar para o objeto e olhar de volta para o avaliador (ou olha primeiro para o objeto, depois para o avaliador e em seguida de volta ao objeto), deixando clara a intenção de indicar com o olhar o objeto que ela quer mostrar e se certificando com o olhar que o avaliador acompanhou o seu olhar compartilhado. Bebês com 9 meses já apresentam atenção compartilhada.

A criança não apresenta nenhum gesto que indique ao avaliador e/ou aos pais/cuidadores o seu interesse por um objeto.	5	
SEM FAZER CONTATO VISUAL a criança puxa o avaliador até o objeto, OU coloca a mão do avaliador em cima do objeto, OU entrega o objeto para o avaliador.	4	
A criança indica o objeto para o avaliador apontando, vocalizando ou não; mas não olha para o avaliador e nem para o objeto.	3	
A criança olha para o objeto do seu interesse, podendo apontar e/ou vocalizar, mas não olha para o avaliador; OU a criança olha em direção ao avaliador enquanto aponta para o objeto, mas não olha para o objeto (ou seja, o olhar não faz a ligação do objeto com o avaliador); OU a criança faz o que está descrito no item "1" apenas uma vez.	2	
A criança olha para o avaliador e depois para o objeto, mas não olha de volta para o avaliador para complementar o ciclo da atenção compartilhada (pode ao mesmo tempo vocalizar e/ou apontar); OU a criança faz o ciclo completo de atenção compartilhada apenas uma vez.	1	
A criança apresenta olhar compartilhado conforme descrito na epígrafe. Pode, ao mesmo tempo, vocalizar e/ou apontar compartilhando com o avaliador o seu interesse pelo objeto.	0	

3.4. Resposta da criança às tentativas do avaliador de atenção compartilhada

Esse item se refere à resposta da criança às iniciativas do avaliador de compartilhar com ela o interesse por algum objeto. A sequência da tarefa é interrompida quando a criança olhar para o objeto indicado: 1. posicione um brinquedo (o ursinho) a certa distância da criança (forme um triângulo entre você a criança e o objeto), chame-a pelo nome e se certifique que ela olha para você, em seguida diga "olhe", dirija o seu olhar em direção ao brinquedo e volte o seu olhar para a criança, indicando através do olhar o seu desejo de que ela olhe para aquele objeto (caso a criança não olhe repita a mesma ação pelo menos três vezes). 2. Repita a mesma ação anterior e diga: "olhe

LABIRINTO
Laboratório Interdisciplinar de Pesquisa em Autismo

fulano aquele ursinho ali" (caso a criança não olhe repita menos três vezes). 3. Repita a mesma ação anterior e também vire o seu rosto de forma exagerada em direção ao objeto (caso a criança não olhe repita pelo menos três vezes). 4. Caso a criança não olhe, repita a mesma ação anterior, agora também apontando com o indicador em direção ao objeto (caso a criança não olhe, repita pelo menos três vezes). 5. Caso a criança mesmo assim não olhe, repita a mesma ação anterior, agora também tocando na criança (caso a criança não olhe, repita pelo menos três vezes). Em qualquer momento que ela olhar para o ursinho, pegue o brinquedo e o ofereça para ela brincar, para que a solicitação não termine no vazio.

A criança não responde a nenhuma tentativa de atenção compartilhada.	5	
A criança olha para o objeto depois que avaliador toca nela, nomeia, vira o rosto de forma exagerada e aponta para o objeto.	4	
A criança olha para o objeto quando o avaliador nomeia, vira o rosto de forma exagerada e aponta para o objeto.	3	
A criança olha para o objeto quando o avaliador nomeia e vira o rosto de forma exagerada em direção ao objeto.	2	
A criança olha para o objeto quando o avaliador além de olhar nomeia o objeto.	1	
A criança acompanha o olhar do avaliador e olha para o objeto em umas das três primeiras tentativas.	0	

3.5. Gestos Comunicativos

Considerar nesse item os gestos que tenham intenção comunicativa, sejam auxiliares à comunicação verbal ou substitutos, exemplo: dar tchau, legal, gesto que indica comer (mexe a mão perto da boca), pare (abre a palma da mão em direção ao interlocutor), onde está? (mãos viradas para baixo), silêncio (dedo na boca), dormir (cabeça encostada nas mãos), vem cá (movimenta os dedos da mão), senta aqui (mão no chão indicando local), ninar nenê (balançar braços cruzados), gesto de indicando sim e não, indicar números com os dedos, etc. Se o único gesto for bater palmas, verificar se não se trata de maneirismo.

A criança não apresenta nenhum gesto comunicativo.	5	
A ÚNICA forma de comunicação não verbal da criança é apontar de forma vaga, com o braço ou com a mão, ao invés de apontar com o indicador para mostrar algo distante.	4	
A criança usa gestos comunicativos apenas imitando (exemplo: o entrevistador dá tchau, ou joga um beijo e ele imita); OU APENAS usa o dedo indicador para apontar espontaneamente para mostrar algo distante.	3	
A criança só usa gestos comunicativos quando solicitada a fazê-lo ("jogue um beijo", "dê tchau", "mostre nos dedos quantos anos você tem"), E/OU os gestos são mecânicos ou diferentes dos padrões socioculturais habituais (por exemplo: dá tchau com o dorso da mão, com a mão fechada).	2	
A criança se comunica espontaneamente com gestos conforme descrito no item "0", porém é menos frequente do que seria esperado para uma criança da sua idade.	1	
A criança se comunica por gestos de forma espontânea, usando gestos como auxiliares ou substitutos da comunicação verbal.	0	

LABIRINTO
Laboratório Interdisciplinar de Pesquisa em Autismo

4. Comportamento rígido, estereotipado e maneirismos

4.1. Brincar/simbolização

Esse item se refere à capacidade de criança de acesso ao mundo simbólico, nessa faixa de idade verificado através da forma como ela brinca. O acesso ao simbólico se opõe ao brincar repetitivo e estereotipado, característico de crianças com autismo. ***O brincar funcional simples*** *se refere ao uso apropriado do brinquedo (sacodir o chocalho, jogar a bola, ninar a boneca, arrastar o carrinho). O* ***brincar funcional composto*** *é uma associação convencional de dois ou mais brinquedos (dar a mamadeira à boneca, mexer a panela com o talher).* ***O brincar simbólico*** *ou faz-de-contas implica na atribuição de propriedades que não são inerentes ao objeto (usar um lápis como se fosse uma colher, um bloco como se fosse comida), ou atribuir presença a objetos/situações imaginadas (fingir que queimou a mão na panela quente, fingir que come algo no prato vazio, ou bebe algo no copo vazio).* ***O brincar estereotipado*** *se refere a um padrão ritualístico e perseverante de uso dos brinquedos ou outros objetos (alinhar brinquedos, colocar e tirar brinquedos de algum recipiente, brincar com partes de brinquedos, separar brinquedos por cores, formas ou tamanho, girar os brinquedos ou parte deles, deixar os brinquedos caírem de certa altura, etc.). Crianças entre 2 e 3 anos brincam de forma funcional com vários brinquedos; crianças de 3 a 4 anos já começam a fazer brincadeiras simbólicas espontâneas (faz de contas); crianças de 4 a 5 anos já tem uma grande capacidade de fantasiar.*

A criança nunca usa os brinquedos OU apenas arremessa os brinquedos no chão de forma desordenada, e/ou apenas os cheira ou os coloca na boca.	5	
A criança usa os brinquedos APENAS de forma estereotipada.	4	
A criança RARAMENTE (uma ou duas vezes) brinca de forma funcional simples. Brinca, na MAIOR PARTE DO TEMPO, de forma estereotipada.	3	
A criança faz brincadeiras funcionais simples e RARAMENTE (um ou duas vezes) faz brincadeiras funcionais compostas. Quando ocorre brincar simbólico é imitando o avaliador OU cenas fixas. Pode TAMBÉM brincar de forma estereotipada.	2	
Brinca funcionalmente (simples e composto) com os brinquedos e realiza algumas brincadeiras simbólicas. O brincar é menos flexível e criativo do que seria esperado para uma criança da sua idade.	1	
Brinca funcionalmente com vários brinquedos e realiza brincadeiras simbólicas espontâneas, de forma flexível e criativa, compatível com a sua idade.	0	

4.2. Dificuldade de mudança e interesses restritos.

Esse item se refere a comportamentos que expressam que a criança resiste à troca do brinquedo/objeto ou da atividade (ex. desenhar, usar o celular). Pode também resistir às propostas de mudança feitas pelo avaliador na forma como ela usa o objeto ou realiza uma atividade. A dificuldade de mudança pode se revelar por interesses restritos, com a adesão da criança a alguma atividade, ou brinquedo/objeto.

A criança fica aderida a algum brinquedo/objeto ou atividade, do qual não aceita se separar. Resiste intensamente a qualquer tentativa de retirar o brinquedo/objeto ou mudar a atividade.	5	
Apresenta interesse muito forte por algum brinquedo/objeto ou atividade. Resiste às tentativas de retirar o brinquedo/objeto ou mudar a atividade; mas busca ou aceita APENAS UM outro brinquedo durante a avaliação.	4	
Apresenta interesse maior por algum brinquedo/objeto ou atividade. Resiste às tentativas de retirar o brinquedo/objeto ou mudar a atividade; mas busca ou aceita pelo menos DOIS	3	

outros brinquedos durante a avaliação.		
Apresenta nítida preferência por algum brinquedo/objeto ou atividade, ao qual volta com frequência de pelo menos DUAS VEZES, mas não resiste às propostas de mudanças de brinquedos ou atividades.	2	
A criança nota que houve mudança do brinquedo ou atividade, pode retornar à mesma atividade ou buscar o mesmo brinquedo UMA VEZ, mas não resiste às mudanças de brinquedos ou atividades.	1	
A criança não resiste à mudança nos brinquedos e atividades durante a avaliação.	0	

4.3. Maneirismos.

Referem-se a movimentos repetitivos e ritmados, que são executados sem uma finalidade e são intencionais (ex: balançar o corpo ou a cabeça, balançar as mãos (flapping), estalar os dedos, bater as mãos, montar os dedos uns em cima dos outros, girar punhos etc. Não cotar aqui movimentos que claramente estão associados a hipo ou hiper reatividade sensorial, que devem ser cotados no item 4.4.

A criança apresenta maneirismos conforme descritos acima A MAIOR PARTE DO TEMPO durante a avaliação.	5	
A criança apresenta maneirismos conforme descritos acima em VÁRIAS (mais de cinco) ocasiões durante a avaliação, mas não a maior parte do tempo.	4	
A criança apresenta maneirismos conforme descritos acima em ALGUMAS (de três a cinco) ocasiões durante a avaliação.	3	
A criança apresenta maneirismos conforme descritos acima RARAMENTE (uma ou duas ocasiões) durante a avaliação.	2	
A criança JÁ APRESENTOU maneirismos conforme descritos acima, ou apresenta raramente, de modo que os pais referem, mas não foram observados durante a avaliação.	1	
A criança NUNCA apresentou maneirismos conforme descritos acima	0	

4.4. Comportamentos de hipo ou híper reatividade sensorial.

Reatividade Sensorial é um padrão de respostas às experiências sensoriais, observadas através de comportamentos que se manifestam como hiper reatividade (reações exageradas/excessivas) ou hipo reatividade (reações ausentes/reduzidas) a informações sensoriais do ambiente ou do próprio corpo, quando comparadas com crianças com desenvolvimento típico. Por exemplo: não perceber quando tocado, parecer não notar que está sujo, pisar em objetos pelo chão, esfregar-se no chão, morder camisa, cheirar ou lamber objetos, passar brinquedos no corpo, olhar brinquedos de ângulos diferentes (ex. deitar-se no chão e movimentar o carrinho diante dos olhos), fixar-se no movimento ou sons dos brinquedos, pular constantemente, andar constantemente de um lado para o outro, andar na ponta dos pés. Inclui também incômodo com som (coloca as mãos nos ouvidos diante de alguns sons), com texturas, com o toque (afastar-se quando tocado), com movimento (quando adulto pega no colo ou faz brincadeiras de jogar pra cima ou aviãozinho) etc.
OBS: Para identificar com maior precisão os aspectos sensoriais, os pais devem responder o questionário sensorial.

A criança apresenta alterações na reatividade sensorial conforme descrito acima A MAIOR PARTE DO TEMPO.	5	
A criança apresenta alterações na reatividade sensorial conforme descrito acima em VÁRIAS ocasiões (mais de cinco vezes), mas não a maior parte do tempo.	4	

LABIRINTO
Laboratório Interdisciplinar de Pesquisa em Autismo

A criança apresenta alterações na reatividade sensorial conforme descrito acima em ALGUMAS ocasiões (de três a cinco vezes).	3	
A criança apresenta alguma alteração na reatividade sensorial descrita acima de forma clara em um ou dois momentos durante a avaliação. Apesar de ocorrerem durante a avaliação, ocorrem RARAMENTE.	2	
A criança JÁ APRESENTOU alguma alteração na reatividade sensorial descrita acima, ou apresenta raramente, de modo que os pais referem, mas não foram observados durante a avaliação.	1	
A criança nunca apresentou alteração na reatividade sensorial.	0	

Para mais informações acesse: www.congressolabirinto.com.br
